# *Escherichia coli* high-risk clone ST410 harboring *bla*_NDM-13_ isolated from hospital wastewater in China

**DOI:** 10.1007/s11356-023-28193-6

**Published:** 2023-07-21

**Authors:** Xiaoyang Ju, Yuchen Wu, Gongxiang Chen, Rong Zhang

**Affiliations:** Department of Clinical Laboratory, The Second Affiliated Hospital Zhejiang University School of Medicine, Hangzhou, 310009 China

**Keywords:** *bl*a_NDM-13_, Hospital wastewater, ST410 *E. coli*, Antibiotic resistance

## Abstract

A carbapenem-resistant *Escherichia coli* strain C-SRM-3 was isolated from hospital wastewater effluent in Hangzhou city, China in March 2022. Analysis of the whole genome sequence showed that this *bla*_NDM-13_-positive strain belonged to an internationally recognized high-risk clone ST410 responsible for the dissemination of carbapenem resistance in *E. coli*. This isolate displayed a multidrug-resistant phenotype and carried a cassette of antibiotic-resistant genes. *bla*_NDM-13_ gene was successfully transferred to the recipient *E. coli* C600 via conjugation. WGS results revealed that *bla*_NDM-13_ gene was located on an IncI1 type plasmid replicon. The phylogenetic reconstruction showed that wastewater-sourced C-SRM-3 strain was located in a single branch, far removed from human-derived and animal-sourced isolates. The detection of *bla*_NDM-13_ in hospital wastewater suggests that continuous monitoring of antibiotic-resistant genes in the environment is critical for the prevention of carbapenem-resistant bacteria spreading.

**Supplementary Information:**

The online version contains supplementary material available at 10.1007/s11356-023-28193-6.

Carbapenemase producing organisms (CPO) is a major threat to the global public health because these bacteria were resistant to almost all clinically available β-lactam antibiotics and therefore greatly limit the therapeutic options (Bonomo et al. 2018). New Delhi metallo-β-lactamase (NDM) is one of the most common carbapenemases worldwide and was firstly discovered in 2009 (Yong et al. 2009). Up to now, more than twenty NDM variants have been identified in multiple bacterial species (Wu et al. 2019). NDM-13 contains two amino acid substitutions (D^95^N and M^154^L) compared with NDM-1, but the two NDM variants have similar enzymatic activity profiles towards β-lactams (Shrestha et al. 2015).

To our knowledge, *bla*_NDM-13_ has been reported from clinical samples in only three countries, including *E. coli* from urine in Nepal (Shrestha et al. 2015), *E. coli* from rectal swab sample in Korea (Kim et al. 2019), and *E. coli* and *Salmonella* Rissen isolated from urine and fecal sample in China (Huang et al. 2022; Lv et al. 2016). No report on the emergence of *bla*_NDM-13_ gene strains isolated from the environment has been published. Hospital wastewater is regarded as an emerging pollutant. Multidrug-resistant pathogens and residual antibiotics in hospital wastewater can enter into the water system in the broader environment and thus increase the potential risk of spreading resistance in aquatic environment (Hocquet et al. 2016). In this study, we described the molecular characterization of an NDM-13-producing *E. coli* obtained from hospital sewage in China.

The strain C-SRM-3 was isolated from the wastewater effluent of a tertiary comprehensive hospital in Hangzhou city, China in March 2022. Both NG-test CARBA5 and GeneXpert system confirmed the presence of NDM. Conjugation experiments were carried out using filter mating method, with rifamycin-resistant *E. coli* C600 strain as the recipient. The conjugants were randomly selected on Mueller–Hinton agar plates containing 600 µg/mL rifampicin and 2 µg/mL meropenem and then inoculated on the selective media Chinablue plates at 37 °C for 18 h. Based on the morphological distinction, the donor C-SRM-3 showed a blue phenotype on the Chinablue plate, while the recipient EC600 was red. Red clones were picked for further PCR assays and whole genome sequencing to verify the success of the horizontal transfer of *bla*_NDM-13_ gene. In our experiment, *bla*_NDM-13_ gene was successfully transferred to the recipient strain EC600.

Antimicrobial susceptibility of strain C-SRM-3 and its conjugant C-SRM-3JH were determined by the microbroth dilution method. The MICs of most antibiotics were interpreted according to Clinical and Laboratory Standards Institute (CLSI) guidelines, and the results of tigecycline were interpreted based on EUCAST standards (Version 12.0, 2022). Isolate C-SRM-3 displayed phenotypic resistance to imipenem, meropenem, ertapenem, cefmetazole, ceftazidime, ceftazidime, cefotaxime, ceftazidime/avibactam, piperacillin/tazobactam, cefoperazone/sulbactam, cefepime, ciprofloxacin, aztreonam, but was susceptible to polymyxin B, tigecycline and amikacin (Table 1).

**Table 1:** Antibiotic susceptibility profiles of strain C-SRM-3, conjugant C-SRM-3-JH and recipient EC600

Antibiotics	Breakpoint for resistance (μg/ml)	C-SRM-3	C-SRM-3-JH	EC600
Imipenem	≥ 4	**4**	**8**	≤ 1
Meropenem	≥ 4	**4**	**4**	≤ 1
Ertapenem	≥ 2	**8**	**8**	≤ 2
Cefmetazole	≥ 64	**64**	**16**	≤ 2
Ceftazidime	≥ 16	** > 128**	** > 128**	≤ 2
Cefotaxime	≥ 4	** > 128**	**128**	≤ 4
Piperacillin/Tazobactam	≥ 128/4	**256/128**	**128/4**	≤ 8/4
Cefoperazone/Sulbactam	≥ 64/32	** > 256/128**	** > 256/128**	≤ 8/4
Ceftazidime/Avibactam	≥ 16/4	** > 64/4**	** > 64/4**	≤ 0.5/4
Cefepime	≥ 16	**64**	**32**	≤ 4
Polymyxin B	≥ 4	1	≤ 0.5	≤ 0.5
Tigecycline	≥ 0.5	≤ 0.25	≤ 0.25	≤ 0.25
Ciprofloxacin	≥ 1	** > 32**	≤ 1	≤ 1
Amikacin	≥ 64	≤ 4	≤ 4	≤ 4
Aztreonam	≥ 16	**128**	≤ 4	≤ 4

Resistant phenotypes are bolded

To characterize the molecular features of *E. coli* C-SRM-3, whole genome sequencing (WGS) was performed using the Illumina NovaSeq PE150 and Nanopore MinION (Oxford, UK) sequencing platforms. The short-read sequences were assembled using SPAdes v3.15.1. Hybrid assembly of both sequencing reads was constructed using Unicycler v.0.4.4. C-SRM-3 strain is 5.1 Mb in size. It consists of six circular DNA sequences, including a chromosome of 4,799,348 bp with 50.66% GC content, and five plasmids. The complete plasmid sequence was annotated via RAST Server (https://rast.nmpdr.org/) and edited manually. Circular maps of plasmids were plotted using the BLAST Ring Image Generator (BRIG) tool (Alikhan et al. 2011). Further bioinformatics analysis was performed at the Center for Genomic Epidemiology (www.genomicepidemiology.org) with the bacteria pipeline (ResFinder version 4.1, PlasmidFinder version 2.1 and VirulenceFinder version 2.0). Insertion sequences (ISs) were identified by ISfinder (Siguier et al. 2006). In C-SRM-3, only three plasmids carried antibiotic resistance genes (ARGs). *bla*_NDM-13_ gene, together with the aminoglycosides [*aph*(3*’*)*-Ia, aadA*2], sulphonamides (*sul1*), trimethoprim (*dfrA12*) and disinfectant resistance gene (*qacE*), was located on a 110 kb IncI1-I(Alpha) type plasmid, designated by pCSRM3-NDM13 (Fig. 1). This plasmid has been identified to be a mobile plasmid, and could be transferred into the recipient EC600 strain through conjugation, conferring resistance to multiple antimicrobial agents (Table 1). By BLASTN analysis, three plasmids showed > 80% coverage and > 99% identity with pCSRM3-NDM13, namely p2_025970-F (CP036181.1), pEC6563-NDM5 (CP095858.1) and p1108-emrB (MG825377.1). A circular comparison showed that these plasmids shared the same backbone region, but slight differences among surrounding regions of *bla*_NDM-13_ gene.

**Fig. 1:**
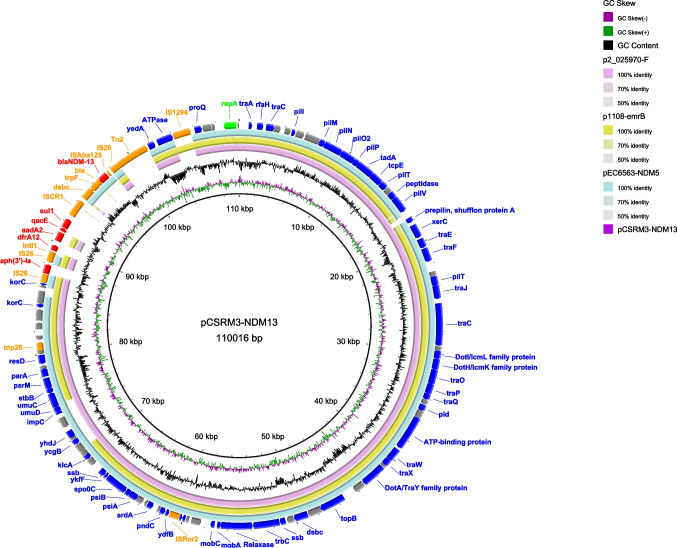
Comparative analysis of pSRM3-NDM13 (this study) with p2_025970-F (CP036181.1), pEC6563-NDM5 (CP095858.1) and p1108-emrB (MG825377.1) plasmids. The outermost circle in the figure represents the pSRM3-NDM13 sequence with gene annotations. In the outermost circle, the red arrows, orange arrows, green arrows, blue arrows and grey arrows indicate ARGs, mobile genetic elements, repA, other genes and hypothetical proteins, respectively

Then, the sequences of bacterial genome carrying *bla*_NDM-13_ were compared and visualized by EasyFig v.2.2.5. ΔIS*Aba125*-*bla*_NDM-13_-*ble*_MBL_-*trpF* was found in all sequences (Fig. S1). Various insertion sequences (IS) such as Tn*2*, IS*26*, IS*CR1*, IS*1294*, IS*5*, IS*3000* were distributed at the upstream or downstream of that region, and probably related to the mobilization mechanism of *bla*_NDM_ gene according to previous studies (Wu et al. 2019).

In addition, plasmid analysis revealed that there were also two plasmid harboring ARGs in C-SRM-3 strain, a 68 kb IncFIB/IncFIC(FII) type plasmid and a 113 kb IncFII/IncX1 type plasmid. The other two plasmids did not carry resistance genes. Based on the VirulenceFinder 2.0 database, virulence factors encoding adhesion and fimbrial attachment proteins (*fimH*, *fdec*), outer membrane protein (*traT*, *iss*, *iucC*), aerobactin system (*iutA*, *iron*, *sitA*) and colicin V (*cvaC*) were detected in this isolate.

Through the online comparison in PubMLST (https://pubmlst.org), we found that *E. coli* C-SRM-3 belonged to the sequence type 410 (ST410). Due to its multidrug resistance and high transmissibility, ST410 *E. coli* has been described as an emerging international high-risk clone in numerous studies (Nadimpalli et al. 2019; Roer et al. 2018; Schaufler et al. 2016). A global epidemiological survey revealed that ST410 was the most common clone among the carbapenemase-producing *E. coli* population (Peirano et al. 2022). Likewise, another nationwide surveillance of CRE strains in China showed that NDM enzymes were the most frequently reported carbapenemase types among carbapenem-resistant *E. coli*, and that ST410 was the second most common type of NDM-positive *E. coli* strains, after ST167 (Zhang et al. 2017).

To place our sequenced *E. coli* C-SRM-3 isolate into a global context, a collection of isolates with the same species were selected from NCBI Pathogen Detection Database(https://www.ncbi.nlm.nih.gov/pathogens/isolates/#taxgroup_name:%22E.coli%20and%20Shigella%22, accessed on 27 March 2023) and a total of 1773 ST410-*E. coli* genomes were identified using the Achtman seven gene MLST scheme as a query. We picked 538 ST410-*E. coli* genomes carrying *bla*_NDM_ genes and performed phylogenetic analysis of the ST410 lineage based on core-genome alignment using Parsnp v1.2 (Treangen et al. 2014) (Table S1). Notably, the global collection of *bla*_NDM_-positive ST410 lineages was predominantly portrayed by human-derived isolates, and to a lesser extent, by animal-sourced isolates and environmental isolates. The phylogenetic reconstruction showed that 538 ST410 isolates can be roughly divided into two clades and eight sub-clades (Fig. 2). Isolate C-SRM-3 was obtained from hospital wastewater. It was located in a single branch, with long distance from human and animal-sourced isolates. According to SNP analysis, the most closely related to C-SRM-3 was the isolate GCA_014117745.2, recovered from human urine-sourced in Thailand in Asia (537 SNPs differences) (Table S2). In terms of geography, *bla*_NDM_-ST410 *E. coli* strains were widely distributed and have been detected in 31 counties across six continents. Additionally, some strains from sub-cluster have a geographical preference, for example isolates prevailing in the American continent were mostly distributed in clade 3 and clade5, while isolates from Africa were mainly distributed in clade 7.

**Fig. 2:**
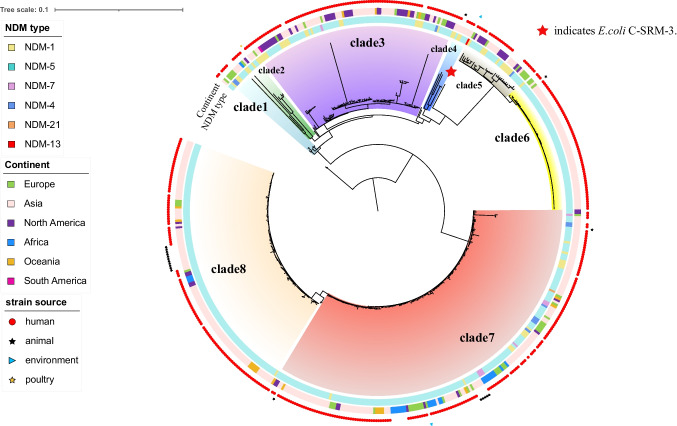
Phylogeny of *bla*_NDM_-positive ST410 *E. coli* genomes (*n* = 538) based on SNP alignment. Red star indicates *E. coli* C-SRM-3

Among the 235964 *E. coli* assembled genomes, only 5203 data were identified as *bla*_NDM_-positive through AMR genotypes search, with a positive rate of 2.20%. However, 547 strains were screened for *bla*_NDM_-positive from 1773 ST410 *E. coli* strains, and the positive rate was 30.85%, which was significantly higher than the average level. It indicates that ST410 *E. coli* served as the major host of *bla*_NDM_-positive *E. coli*, and its clone spread may contribute to the prevalence of *bla*_NDM_ gene among *E. coli* strains. Moreover, the higher positive rate suggests that infections caused by ST410 *E. coli* will be more challenging and other potent antibiotics such as polymyxin and tigecycline should be taken into account in clinical treatment. Figure 2 revealed that six NDM variants had been detected in ST410 *E. coli*, with NDM-5 being the majority and NDM-1, NDM-4, NDM-7, NDM-21 being a small proportion. The presence of *bla*_NDM-13_ in the ST410 *E. coli* further confirmed the spread of high-risk clone ST410 group in environment.

To the best of our knowledge, this is the first report of *bla*_NDM-13_-positive bacteria isolated from hospital wastewater. Hospital wastewater served as a persistent reservoir of pathogens as well as ARGs, and played a crucial role in spreading resistance in the aquatic environment (Zhang et al. 2020). More importantly, high concentrations of antibiotics in the water are recognized as a significant contributor to the horizontal transfer of resistant plasmids, which leads to an increasing prevalence of antibiotic resistant bacteria (Hocquet et al. 2016). Our study provided an insight into the genomic features of high-risk clone ST410 *E. coli* isolate with the *bla*_NDM-13_ from hospital wastewater and highlighted the need to have continuous monitoring of environmental contamination with ARGs. Meanwhile, our study highlighted that proper disposal of hospital wastewater is critical in the prevention of the spreading of carbapenem-resistant bacteria in the environment.

## Supplementary Information

Below is the link to the electronic supplementary material.

Supplementary file1 (XLSX 204 KB)

Supplementary file2 (XLSX 1543 KB)

Supplementary file3 (DOCX 113 KB)

## Data Availability

The complete sequences of *E. coli* C-SRM-3 were deposited in the NCBI database with accession numbers CP123195, CP123196, CP123197, CP123198, CP123199, and CP123200 under the BioSample accession SAMN32472040.
