# Supplementary material for: Escherichia coli high-risk clone ST410 harboring blaNDM-13 isolated from hospital wastewater in China
Source: Environ Sci Pollut Res Int. 2023 Jul 21;30(39):91487–91. doi: 10.1007/s11356-023-28193-6 (PMC10439849; doi:10.1007/s11356-023-28193-6)
Supplement: Supplementary file 3 — Supplementary file3 (DOCX 113 KB) [file 11356_2023_28193_MOESM3_ESM.docx]

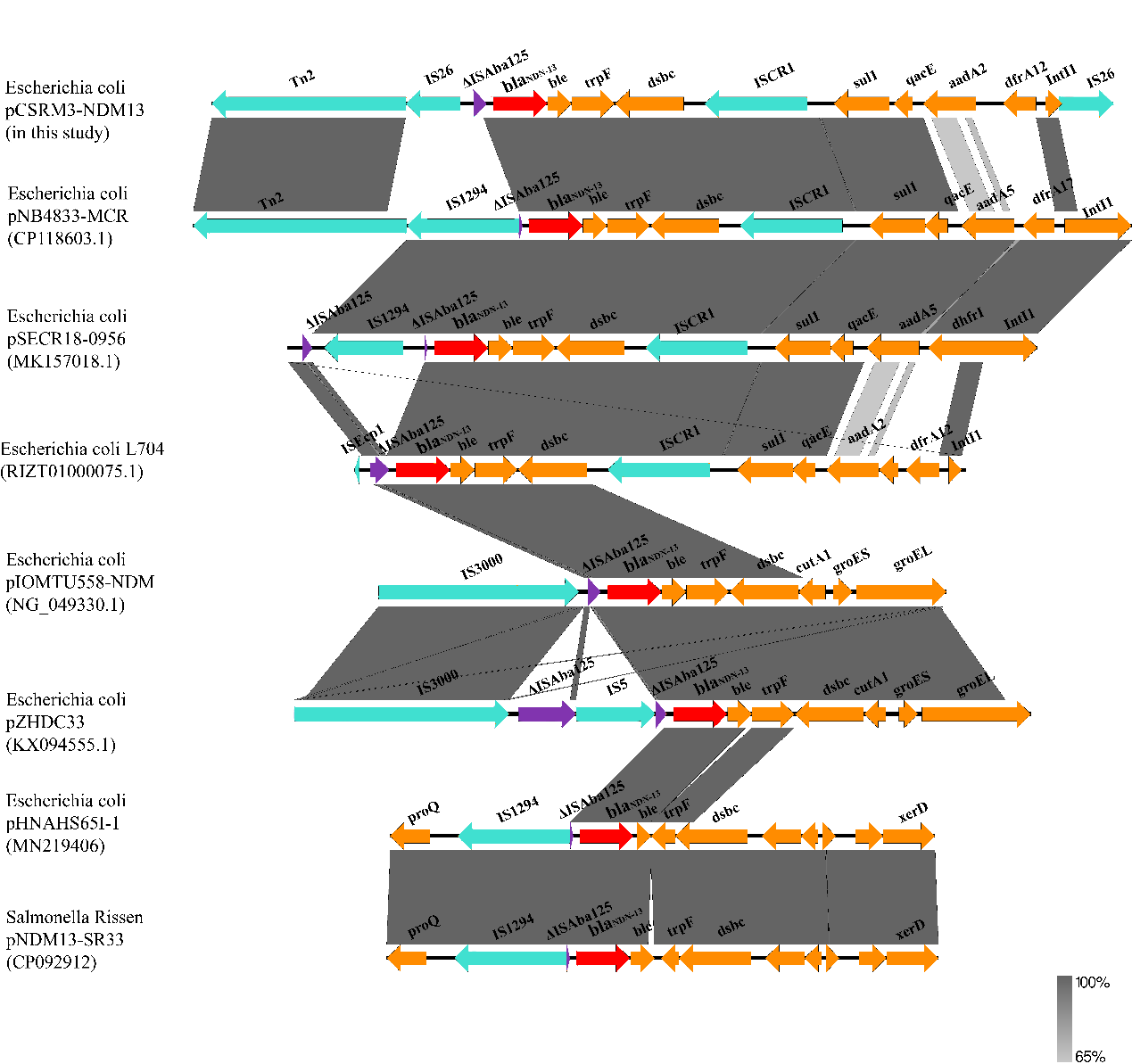


**Figure S1. Comparison of the genetic environment of *bla*_NDM-13_.** Red arrows indicate *bla*_NDM-13_. Purple arrows, blue arrows and orange arrows indicate ΔIS*Aba125*, mobile elements and other proteins, respectively. Grey shading indicates homologous regions.
